# Impaired cortical processing of inspiratory loads in children with chronic respiratory defects

**DOI:** 10.1186/1465-9921-8-61

**Published:** 2007-09-06

**Authors:** Brigitte Fauroux, Francis Renault, Pierre Yves Boelle, Christine Donzel-Raynaud, Frédéric Nicot, Annick Clément, Christian Straus, Thomas Similowski

**Affiliations:** 1 AP-HP, Hôpital Armand Trousseau, Pediatric Pulmonary Department, Paris, 75571 France; 2 Inserm UMR-S 719, Paris, 75000, France, Université Pierre et Marie Curie-Paris6, 75571 France; 3 AP-HP, Hôpital Armand Trousseau, Paediatric Neurophysiology Unit, Paris, 75571 France; 4 AP-HP, Hôpital Saint Antoine, Department of Biostatistics, 75012 Paris, Inserm U444, 75000, Paris, France; 5 AP-HP, Hôpital La Pitié Salpétrière, Respiratory Physiology, Pulmonology and Intensive Care, Paris, France; 6 UPRES EA 2397, Université Pierre et Marie Curie-Paris6, Paris, France

## Abstract

**Background:**

Inspiratory occlusion evoked cortical potentials (the respiratory related-evoked potentials, RREPs) bear witness of the processing of changes in respiratory mechanics by the brain. Their impairment in children having suffered near-fatal asthma supports the hypothesis that relates asthma severity with the ability of the patients to perceive respiratory changes. It is not known whether or not chronic respiratory defects are associated with an alteration in brain processing of inspiratory loads. The aim of the present study was to compare the presence, the latencies and the amplitudes of the P1, N1, P2, and N2 components of the RREPs in children with chronic lung or neuromuscular disease.

**Methods:**

RREPs were recorded in patients with stable asthma (n = 21), cystic fibrosis (n = 32), and neuromuscular disease (n = 16) and in healthy controls (n = 11).

**Results:**

The 4 RREP components were significantly less frequently observed in the 3 groups of patients than in the controls. Within the patient groups, the N1 and the P2 components were significantly less frequently observed in the patients with asthma (16/21 for both components) and cystic fibrosis (20/32 and 14/32) than in the patients with neuromuscular disease (15/16 and 16/16). When present, the latencies and amplitudes of the 4 components were similar in the patients and controls.

**Conclusion:**

Chronic ventilatory defects in children are associated with an impaired cortical processing of afferent respiratory signals.

## Background

Repeated inspiratory occlusions applied at the mouth in humans elicit cortical potentials, usually referred to as respiratory-related evoked potentials (RREPs) [1], which represent one of the respiratory counterparts of somatosensory evoked potentials. These potentials feature a sequence of components reflecting the arrival of impulses at the primary sensory cortex and, for the later components, the cortical processing of the information [1,2]. The first positive component, P1, occurs 40–60 ms after the beginning of the load-related change in mouth pressure. Its amplitude is determined mainly by the physical characteristics of the stimuli [3]. P1 is followed by a negative component N1 (average latency 80 ms), and by later components positive and negative again (P2, and N2). Inspiratory occlusions also elicit later components occurring circa 300 ms after the stimulus which seem to have a cognitive nature [4].

The afferent sources of the RREPs are likely to be multiple and redundant, and mechanoreceptors located within the airways, the lung, the chest wall and the respiratory muscles seem to all contribute to the RREPs. For example, the RREPs are present in bilateral lung transplant recipients, namely in spite of bilateral vagotomy [4]. The upper airways mechanoreceptors probably also play an important role in the generation of the RREPs [5], but these potentials can still be observed in their absence [6]. The RREPs are unaltered by acute inspiratory muscle fatigue [7] and they are present, even though incomplete and abnormal, in patients with severe inspiratory pump dysfunction [8]. Of note, if in such patients, the upper airway is bypassed, the RREPs disappear [8].

Within this frame, chronic abnormalities of the respiratory system, either intrinsic or neuromuscular in origin, could alter the RREPs. The present study tested this hypothesis, by comparing the RREPs in children with asthma, cystic fibrosis (CF), and neuromuscular disease. This question is clinically relevant. Indeed, there are parallels between some characteristics of the RREPs and the ability to perceive inspiratory resistive loads [9] and estimate their magnitude [3,10]. The documentation of the RREPs in patients with respiratory defects could provide an objective estimate of their capacity to detect changes in respiratory mechanics, a capacity that has been shown to have a prognostic value in certain settings. Indeed, in children with a history of life-threatening asthma, inspiratory resistive load detection has been shown to be blunted [11] as it has been observed in adults [12]. Also the aptitude of these patients to estimate the magnitude of inspiratory resistive loads may be impaired [13], and has been shown to be associated with highly abnormal RREPs [14]. We observed also very recently that RREPs components were less frequently observed in children with asthma as compared to healthy pediatric and adult subjects [15]. The aim of the present study was thus to compare RREPs in children with asthma, CF, and neuromuscular disease.

## Methods

### Patients

The study was conducted in agreement with the French regulations and had received appropriate legal and ethical approval (Comité Consultatif de Protection des Personnes se prêtant à des Recherches Biomédicales de l'Hôpital Saint Antoine, Paris, France). The patients and their parents gave written informed consent. The patients were recruited on a consecutive basis from our hospital ward and outpatient clinic, and were included in the study if 1) they had been clinically stable for at least one month; 2) they were able to perform reproducible lung function tests within the 24 hours preceding the study; 3) they had a transcutaneous oxyhemoglobin saturation (SpO_2_) above 90%; and 4) they did not receive any kind of neurotropic substance.

The patients belonged to three categories, neuromuscular disease (n = 16), asthma (n = 21), and CF (n = 32) (Table 1). The group of neuromuscular patients included 10 boys with Duchenne muscular dystrophy, 4 girls with spinal muscular amyotrophy, and 2 girls with Becker muscular dystrophy. Four patients (3 patients with Duchenne muscular dystrophy and one patient with spinal muscular amyotrophy) required nocturnal noninvasive positive pressure ventilation.

**Table 1:** Characteristics of the controls and the patients.

	Controls n = 11	*P *(controls vs pooled patients)	Patients with asthma n = 21	Patients with cystic fibrosis n = 32	Patients with neuromuscular disease n = 16	*P *(between the patient groups)
Male/Female	5/6	NS	12/9	16/16	12/4	NS
Age (years)	12 ± 3	NS	12 ± 3	13 ± 4	12 ± 3	NS
Weight (kg)	51 ± 12	0.008	40 ± 13	37 ± 11#	44 ± 7	< 0.001
Height (cm)	158 ± 18	0.006	144 ± 15	145 ± 18	146 ± 19	NS
FVC (% Pred)	NP	NP	98 ± 15§	67 ± 26	52 ± 22	< 0.0001
FEV_1 _(% Pred)	NP	NP	92 ± 16§	56 ± 28	57 ± 25	< 0.0001
FRC He (% Pred)	NP	NP	105 ± 20	98 ± 24	82 ± 32	NS
FRC Pl (% Pred)	NP	NP	125 ± 20	165 ± 40	NP	< 0.05
SpO_2 _(%)	NP	NP	97 ± 2	94 ± 3	98 ± 1	NS
Rsint (% Pred)	NP	NP	160 ± 21	217 ± 85	183 ± 60	NS

Data are presented as mean ± SD.

Abbreviations: FVC: vital capacity; FEV_1_: forced expiratory volume in one second; FRC He: functional residual capacity by the helium technique; FRC Pl: functional residual capacity measured by plethysmography, SpO_2_: transcutaneous oxyhemoglobin saturation, Rsint: airway resistance by the interruption technique, % Pred: values are expressed as a percentage of predicted value. # height is expressed as the armspan in neuromuscular patients.

The controls were compared to the pooled patients using the Student *t *test or Chi-squared test where appropriate. Comparison between the 3 groups of patients was performed with the Kruskal Wallis rank sum test. #Weight was significantly lower in the patients with cystic fibrosis compared to the other patients. §The asthmatic patients had a significantly better lung function than the two other groups, no significant differences were observed between patients with cystic fibrosis and those with neuromuscular disease. NP: not performed, NS: not significant.

Both the asthma and CF groups were constituted of patients with varying degrees of severity, ranging from "mild" to "severe". For asthma, the "mild" phenotype was defined as a clinical and functional control without inhaled corticosteroids and the "severe" phenotype as the presence of permanent symptoms despite treatment and/or a history of near-fatal asthma. For CF, the "mild" phenotype was defined as a forced expiratory volume in one second (FEV_1_) ≤ 45% predicted and the "severe" phenotype as a FEV_1 _= 45% predicted. The majority of these patients participated to a previous study on RREPs [15].

Eleven healthy children also participated (RREPs measurement only) (Table 1). They were free of any known respiratory or neurological disease.

### Lung function data

Forced vital capacity (FVC), FEV_1_, functional residual capacity, measured by the helium dilution technique (FRC He) and by plethysmography (FRC Pl) were measured. The resistance of the respiratory system (Rrsint) was calculated [16]. SpO_2 _and arterial blood gases were also measured [17,18].

### Respiratory-related evoked potentials

The patients and the controls were instructed to avoid sleep deprivation during the 48 hours preceding the recording of RREPs, and to eat lightly on the study day. They were studied during wakefulness, sitting on a lounge chair with the back, neck and head comfortably supported. They were asked to relax, and wore headphones through which they listened to a quiet musical piece of their choice, in order to mask the auditory ambiance of the laboratory. The children, wearing a noseclip, breathed room air through a flanged mouthpiece and a heated pneumotachograph (Hans Rudolph, Kansas city, MO) connected to a ± 2 cm H_2_O linear pressure transducer (Validyne, Northridge, CA) in order to measure ventilatory flow. Mouth pressure was measured from a side port of the mouthpiece, using a differential pressure transducer (DP 15–32 Validyne). The pneumotachograph was assembled in series to a nonrebreathing two way valve (2600 series, Hans Rudolph) of which the inspiratory port could be occluded by an inflatable balloon (9340 series occlusion valve and 9330 series compressor, Hans Rudolph). The inspiratory airflow detected by the pneumotachograph was transmitted to a command box (Neuroservices, Savigny sur Orge, France) that triggered the occlusion valve. Each occlusion stimulus started 250 ms after the onset of the inspiration defined as flow reversal and lasted 400 ms; the average pressure change was 3.6 ± 0.4 cm H_2_O [15]. Occluded breaths were separated by two to six unoccluded breaths.

Evoked responses to occlusions were recorded using standard surface electrodes placed on the scalp at C_3 _and C_4_, and C_z _according to the international 10–20 system [19]. The ground electrode was placed on the left mastoid process. Electrodes impedances were checked at regular intervals and maintained below 5 kOhms. The signals from left (C_3_-C_z_) and right (C_4_-C_z_) parietal areas were separately recorded from 350 ms before to 400 ms after the occlusion stimulus (Viking Select, Nicolet, Madison, WI). The signal was amplified at a 5000 Hz rate over a 1–250 Hz bandwidth. A given occluded breath was retained for averaging only in the presence of a stable electroencephalographic signal baseline and in the absence of obviously aberrant accidents. Evoked responses to 80 consecutive occlusions were averaged [14].

The RREPs were manually analysed. The presence or absence of the first positive (P1), first negative (N1), second positive (P2), and second negative (N2) components of the RREPs were noted. The latencies and baseline to peak amplitudes of the components were measured according to Davenport *et al*. [14]. Amplitudes were also measured from peak to peak [15].

### Statistical analysis

Right-to-left comparisons of the presence of the various components of the RREPs were conducted within the controls and within each category of patients using the McNemar test. Right-to-left comparisons of the amplitudes and latencies of the various components of the RREPs were conducted within the controls and each category of patients using the Wilcoxon signed rank sum test. The controls were compared to the pooled patients using the Student *t *test or Chi-squared test where appropriate. For each component of the RREPs considered in a dichotomous manner (present vs. absent), the three categories of patients were compared using the Fisher's exact test. For quantitative variables, comparisons between the 3 groups of patients were conducted using the Kruskall-Wallis rank sum test. In all analyses, differences were considered significant when the probability p of a type I error was 5% or less. Values are reported as mean ± SD.

## Results

### Patients and controls

The age and gender distribution were similar in the controls and the 3 groups of patients but the controls had a significantly greater height and weight than the patients (Table 1). Mean weight was significantly lower in the patients with CF compared to the patients with asthma and neuromuscular disease. Mean FVC and FEV_1 _were significantly lower in the patients with CF and neuromuscular diseases than in the patients with asthma. No difference was observed between the 3 categories of patients for mean FRC He, SpO2 and Rsint (Table 1), but FRC Pl was significantly increased in patients with CF compared to the asthmatic patients.

### Presence and characteristics of the RREPs components

**Figure 1:**
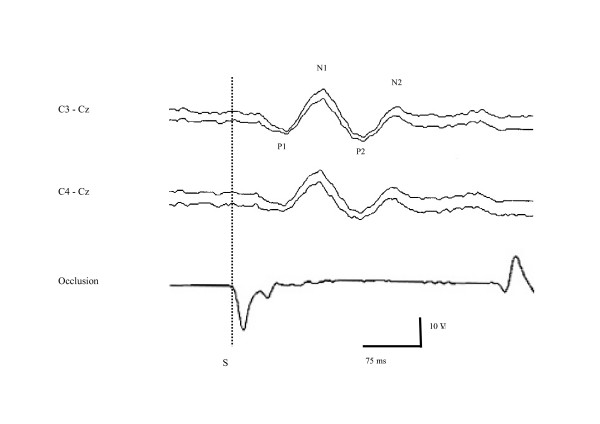
Individual example of a patient with Duchenne muscular dystrophy showing the 4 components of the respiratory related evoked potential following inspiratory airway occlusion. Traces represent from top to bottom: left (C_3_-C_z_) response, right (C_4_-C_z_) response, and real time mouth pressure. S: onset of occlusion stimulus.

All the components of the RREPs were present on both sides in 100% of the control subjects (Table 2), with normal latencies (data not shown) and expected baseline to peak amplitudes (Table 3). Peak to peak amplitudes were also within the normal range (data not shown). An example of RREPs is shown in Figure 1.

**Table 2:** Presence of the different components of the respiratory-related evoked potentials (RREPs) in the controls and the patients with asthma, cystic fibrosis, and neuromuscular diseases.

RREP components	Controls n = 11	*P *(controls vs pooled patients)	Patients with asthma n = 21	Patients with cystic fibrosis n = 32	Patients with neuromuscular disease n = 16	*P *(between the patient groups)
P1 (C_z_-C_3_)	11	0.003	12	14	12	NS
P1 (C_z_-C_4_)	11	0.01	13	17	13	NS
N1 (C_z_-C_3_)	11	0.0002	14	17	16 #	0.07
N1 (C_z_-C_4_)	11	0.02	16	20	15 #	0.002
P2 (C_z_-C_3_)	11	0.0001	13	12	14 #	0.003
P2 (C_z_-C_4_)	11	0.00001	16	14	16 #	0.0001
N2 (C_z_-C_3_)	11	0.004	9	14	8	NS
N2 (C_z_-C_4_)	11	0.003	10	13	10	NS

The controls were compared to the pooled patients using the Student *t *test or Chi-squared test where appropriate. Comparison between the 3 groups of patients was made using the Fisher's exact test. #Significant differences were observed between the patients with neuromuscular disease and the two other groups, no differences were observed between patients with cystic fibrosis and asthma.

**Figure 2:**
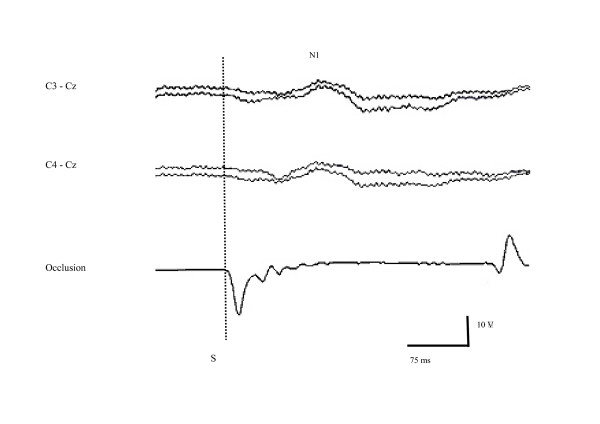
Individual example of a patient with congenital muscular dystrophy of unknown origin showing only the N1 component of the respiratory related evoked potential following inspiratory airway occlusion. The P1 component is not reproducible. Traces represent from top to bottom: left (C_3_-C_z_) response, right (C_4_-C_z_) response, and real time mouth pressure. S: onset of occlusion stimulus.

The 4 components of the RREPs were significantly less frequently observed in the patients than in the controls (Table 2 and Figure 2). Within the 3 groups of patients, the N1 and the P2 components were significantly less frequently observed in the patients with CF than in the patients with asthma or a neuromuscular disease. However, the P1 and the N2 component were observed with a similar frequency in the 3 groups. The complete P1-N1-P2-N2 sequence was observed in only 2 patients with moderate asthma, in 3 patients with CF with moderate lung disease, and in 3 patients with neuromuscular diseases. The RREPs lacked completely in 3 asthmatic patients (which was not significantly different from control subjects and neuromuscular patients) and 12 patients with CF (which was a significantly higher proportion than in any of the other groups, p = 0.012). These 3 asthmatic patients and 12 patients with CF did not differ from the other patients within their group, either with regard to their clinical presentation, or with regard to their lung function.

For all the 4 components, when present, the latencies were similar in the controls and the 3 categories of patients (data not shown).

Table 3 shows the baseline to peak amplitudes of the 4 components. The only significant difference was a greater amplitude of the N1 component in the controls (mean 5.46 ± 3.84 μV) compared to the pooled patients (mean 2.73 ± 1.77 μV, p = 0.02). The peak to peak amplitudes were comparable among the controls and the pooled patients, and within the 3 patient groups (data not shown).

**Table 3:** Baseline to peak amplitudes of the different components of the respiratory-related evoked potentials (RREPs) in the controls and the patients with asthma, cystic fibrosis, neuromuscular diseases.

RREPs component amplitudes (μV)	Controls n = 11	*P *(controls vs pooled patients)	Patients with asthma n = 21	Patients with cystic fibrosis n = 32	Patients with neuromuscular diseases n = 16	*P *(between the patient groups)
P1 (C_z_-C_3_)	3.07 ± 2.07	NS	0.94 ± 0.61	1.54 ± 1.11	1.81 ± 1.28	NS
P1 (C_z_-C_4_)	3.07 ± 2.07	NS	1.12 ± 0.72	1.25 ± 0.85	1.21 ± 0.72	NS
N1 (C_z_-C_3_)	5.46 ± 3.84	0.03	2.21 ± 1.48	3.00 ± 2.51	3.83 ± 2.39	NS
N1 (C_z_-C_4_)	5.46 ± 3.84	0.02	2.02 ± 1.13	2.97 ± 2.28	3.21 ± 1.90	NS
P2 (C_z_-C_3_)	2.15 ± 1.57	NS	1.25 ± 1.42	2.10 ± 1.54	3.00 ± 1.90	NS
P2 (C_z_-C_4_)	2.15 ± 1.57	NS	1.54 ± 1.21	1.81 ± 1.14	1.33 ± 0.82	NS
N2 (C_z_-C_3_)	2.39 ± 1.42	NS	1.11 ± 0.75	1.64 ± 0.87	2.75 ± 2.05	NS
N2 (C_z_-C_4_)	2.39 ± 1.42	NS	2.17 ± 1.53	1.75 ± 1.11	2.91 ± 2.36	NS

Data are presented as mean ± DS. The controls were compared to the pooled patients using the Student *t *test or Chi-squared test where appropriate Comparison between the 3 patient groups was made using the Kruskal Wallis rank sum test.

No qualitative or quantitative right-to-left difference was detected (Table 2).

## Discussion

The four main components of the RREPs were significantly less frequent in children with stable chronic lung and airway diseases or with a ventilatory defect of neuromuscular origin than in age-matched healthy controls. This suggests that chronic ventilatory defects in children can be associated with an impaired cortical processing of afferent respiratory signals. In addition, the RREPs abnormalities were more pronounced in asthmatic children and in children with CF than in children with a ventilatory defect due to a neuromuscular disease. Indeed, the N1 and P2 components were significantly less frequently seen in the patients with asthma or CF, and the number of patients exhibiting no RREPs at all was significantly greater in the CF group.

### Physiological considerations

The CF patients enrolled in this study were comparable to those evaluated in previous studies [20,21]. The respiratory mechanics of these patients is characterized by an increase in the work of breathing as assessed by the high values of inspiratory and diaphragmatic pressure time products. This elevated work of breathing is the signature of a chronically augmented mechanical load, with an elastic component attested by the presence of dynamic hyperinflation and a resistive component in line with the chronic, irreversible, airflow obstruction. Breathing against an elevated background resistance increases the threshold for detection of an added resistance [22]. Yet it has been shown that this also reduces the amplitude of the RREPs elicited by the sudden application of a resistive load [6]. In fact, RREPs elicited by an additional resistance are present only if this resistance exceeds the value of the detection threshold [6]. The increase in background load could thus explain why the RREPs were more abnormal in the CF patients than in the other patient groups. However, we did not study resistance-elicited RREPs, but rather occlusion-induced ones. By definition, and except if the capacity to detect a resistance is totally abolished, the infinite resistance that corresponds to an inspiratory occlusion should be above the detection threshold and thus still elicit a RREP even in the presence of an increased background resistance [6]. This has however not been studied specifically, and how a very high load sustained indefinitely as in our CF patients can modify the RREP threshold as compared to a resistance detection threshold is not known. Of note, and even though no statistically significant trend was detected, some of the CF patients who completely missed the RREPs were among the patients with the most severe respiratory mechanics and respiratory muscle abnormalities (data not shown). That the RREPs abnormalities observed were dichotomous (absence of components, but normal latencies and amplitudes of the preserved components) seems to support the role of the background load. We however acknowledge that this hypothesis would be have been much strengthened if we had demonstrated that our CF patients were less apt than the other patients to detect added inspiratory loads. We did not assess this point, and to our knowledge, there is currently insufficient data to support this hypothesis.

Patients with neuromuscular weakness but without overt respiratory mechanics abnormalities do not breathe against increased loads. This is also the case for asthmatic patients with reversible airway obstruction when they are in clinical remission, which was the case for all the asthmatic patients in the present study. Nevertheless we observed RREPs abnormalities in these two categories of patients. The "increased background resistance" mechanism is therefore probably not the unique one to call upon to explain our findings.

Technical explanations, such as differences in skin impedance between control subjects and sick patients in general, and between CF patients and others in particular, can be ruled out because we paid careful attention to check and maintain the impedance of our recording montage as low as possible in all cases. From a technical point of view, a greater incidence of the N1 peak than the P1 peak may be surprising for a pattern of neural activity that has been reported previously to occur as a linked sequence of neural events. This may be the result of our exclusive use of the C_z _reference electrode montage. Indeed, the P1 peak dipole is best modelled in the somatosensory cortex recorded with electrodes placed 2 cm caudal to the C_3 _and C_4 _sites [23]. The N1 peak is best modelled at the C_z _site using a non-cephalic reference. Our montage was nevertheless able to identify normal RREPs with the full sequence of components in healthy subjects.

It must also be noted that we did not perform the respiratory mechanics and respiratory muscles measurements in the patients with asthma or a neuromuscular disease. The absence of abnormalities in these patients is therefore only assumed, and we may thus have missed an increase in inspiratory load in some of the participants. Beside this possibility, patients with weak inspiratory muscles faced to an inspiratory occlusion develop an inspiratory effort that is closer to their maximal inspiratory output than patients exhibiting normal muscle strength. It cannot be excluded that this interferes in some way with the production of the RREPs.

The role of inflammation must also be discussed. Patients with asthma and CF characteristically exhibit airway inflammation in the broadest sense of the term, although the pathophysiology and the course of the inflammatory process differ between the two diseases. In CF, airway inflammation occurs early in life, is poorly controlled by anti-inflammatory drugs, and is closely linked to bacterial airway infection [24]. In asthma, inflammation can occur at different ages, can be efficaciously controlled by inhaled corticosteroids, and is not linked to bacterial airway infection [25]. As a result, asthma and CF have radically different clinical and functional profiles. Schematically, asthma can be viewed as a chronic disease featuring episodes of reversible airway obstruction of various magnitudes. CF is a chronic lung disease characterised by a chronic and progressively worsening airway obstruction. Hyperinflation is early and constant in CF [20,26], but it is observed only in the most severe asthmatic patients experiencing poor therapeutic control [27]. Our observation of greater RREPs abnormalities in CF and asthma children than in children with neuromuscular disease may suggest that chronic respiratory inflammation may interfere with the processing of respiratory afferent information by the brain, whatever the underlying mechanism. Indeed the number of CF patients exhibiting no RREP at all was significantly higher than in any of the other patient groups studied. Of note, data in the literature indicate that there may be a relationship between the brain and airway inflammation [28]. Indeed, it has been shown in patients with allergic asthma that some brain regions may be hyperresponsive to disease specific emotional and afferent physiological signals, which may contribute to the dysregulation of peripheral processes, such as airway inflammation [28].

### Clinical perspectives

Our results are not out of line with the observations made in asthmatic children by Davenport *et al*. [14]. These researchers observed that the P1 peak was absent in 6 of 11 patients with life-threatening asthma, whereas it was present in 14 of the 15 control asthmatic patients and in all the 14 nonasthmatic patients. Some of the asthmatic patients included in our study had a history of near-fatal asthma (n = 7), and among them 43% and 57% lacked the P1 component on the right and the left side, respectively, which fits with the 54% proportion reported by Davenport *et al*. [14]. In our study, the presence of RREPs abnormalities in the asthmatic children having no history of near-fatal asthma does not agree with the results of Davenport *et al*. [14], but this may be explained by different population characteristics. To the best of our knowledge, our study is the first to provide RREPs data in children with CF and with a restrictive ventilatory defect of neuromuscular origin.

Davenport *et al*. [14] interpreted their findings within the frame of the blunted perception of inspiratory load that has been observed in asthmatic patients with a history of life-threatening asthma, be they adults [12] or children [11] for load detection [13] or for load magnitude estimation. Subjacent to this interpretation is the temptation to use the RREPs as a possible prognostic tool, for example to identify patients at risk of sudden, unexpected acute exacerbations of their disease without recognising them. Clearly the gap to bridge before this can become a reality is very wide at present. Specifically designed clinical studies are needed. Our data however open the possibility that this concept could apply not only to asthma, but also to other chronic inflammatory respiratory disorders in children. The first step to take in the direction of a clinical use of the RREPs would be, as discussed above, seems to put this neurophysiological information in the perspective of a description of the load detection and load magnitude estimation performances in patients with CF and neuromuscular disease. Then putative correlates with clinical profiles could be sought.

## Conclusion

An impaired cortical processing of afferent respiratory signals, as assessed by the analysis of RREPs, may be observed in children with chronic ventilatory defects.

## Competing interests

The author(s) declare that they have no competing interests.

## Authors' contributions

BF and TS designed the study and wrote the first and final version of the manuscript.

CDR, CS and TS helped BF, FR and FN to set up the RREP analysis in Armand Trousseau Hospital.

BF and AC recruited the patients. BF, FR and FN studied the patients.

PYB performed the statistical analysis

All the authors discussed the data, they all gave their input to the manuscript, contributed to the writing and approved the final version.

## Funding

Brigitte Fauroux was supported by the Assistance Publique – Hôpitaux de Paris, INSERM, Université Pierre et Marie Curie Paris6, Vaincre La Mucoviscidose (VLM) and the Association Française contre les Myopathies (AFM). Frédéric Nicot was supported by Vaincre la Mucoviscidose (VLM). Christine Donzel-Raynaud was supported by a grant from the Centre d'Assistance Respiratoire à Domicile d'Ile-de-France (CARDIF). Thomas Similowski and Annick Clément were supported by a grant "Legs Poix triennal" from the Chancellerie de l'Université de Paris.
