# Correction: Impaired cortical processing of inspiratory loads in children with chronic respiratory defects

**DOI:** 10.1186/1465-9921-9-22

**Published:** 2008-02-23

**Authors:** Brigitte Fauroux, Francis Renault, Pierre Yves Boelle, Christine Donzel-Raynaud, Frédéric Nicot, Annick Clément, Christian Straus, Thomas Similowski

**Affiliations:** 1 AP-HP, Hôpital Armand Trousseau, Pediatric Pulmonary Department, Paris, 75571, France; 2 Inserm UMR-S 719, Paris, 75000, France, Université Pierre et Marie Curie-Paris, 75571, France; 3 AP-HP, Hôpital Armand Trousseau, Pediatric Neurophysiology Department, Paris, 75571, France; 4 AP-HP, Hôpital Saint Antoine, Department of Biostatistics, 75012 Paris, Inserm U444, 75000, Paris, France; 5 AP-HP, Hôpital La Pitié Salpétrière, Respiratory Physiology, Pulmonology and Intensive Care, Paris, France; 6 UPRES EA 2397, Université Pierre et Marie Curie-Paris, Paris, France; 7 Pediatric Pulmonary Department and Research Unit Inserm UMR-S 719, AP-HP, Hôpital d'Enfants Armand Trousseau, 26 avenue du Docteur Arnold Netter, 75571 Paris Cedex 12, France

We would like to make it clear that part of the cohort of patients used in our study [1] was also analysed in an article published in *Clinical Neurophysiology *[2]. Although this study was cited in the original version of our article as reference 15, we feel that the overlap between the two studies should be made plain.
